# Efficacy and Drawbacks of Single-Anastomosis Duodeno-Ileal Bypass After Sleeve Gastrectomy in a Tertiary Referral Bariatric Center

**DOI:** 10.1007/s11695-021-05323-y

**Published:** 2021-04-09

**Authors:** Arnaud Liagre, Francesco Martini, Yves Anduze, Hubert Boudrie, Olivier Van Haverbeke, Stefano Valabrega, Radwan Kassir, Tarek Debs, Niccolò Petrucciani

**Affiliations:** 1 Bariatric Surgery Unit, Clinique des Cedres, Ramsay Générale de Santé, Cornebarrieu, France; 2 Department of Medical and Surgical Sciences and Translational Medicine, Faculty of Medicine and Psychology, St Andrea Hospital, Sapienza University, Via di Grottarossa 1035-9, 00189 Rome, Italy; 3 Department of Digestive Surgery, CHU Félix Guyon, Saint Denis, La Réunion France; 4 Division of Digestive Surgery and Liver Transplantation, Archet II Hospital, University of Nice-Sophia-Antipolis, Nice, France

**Keywords:** Bariatric surgery, Single-anastomosis duodeno-ileal bypass, Sleeve gastrectomy, Complications, Obesity, Revisional surgery

## Abstract

**Background:**

The need for revisional procedures after sleeve gastrectomy (SG) for insufficient weight loss or weight regain, gastroesophageal reflux, or other complications is reported to be 18–36% in studies with 10-year follow-up. Single-anastomosis duodeno-ileal bypass (SADI) may be performed as a revisional procedure after SG. This study aims to evaluate the short- and mid-term outcomes of SADI after SG in a referral center for bariatric surgery.

**Materials and Methods:**

Data of patients who underwent SADI between March 2015 and March 2020 were collected prospectively and analyzed retrospectively. Follow-up comprised clinical and biochemical assessment at 1, 3, 6, 12, 18, and 24 months postoperatively, and once a year thereafter.

**Results:**

Overall, 106 patients underwent SADI after a previous SG. The timeframe between SG and SADI was 50 ± 31.3 months. Postoperative mortality was observed in two cases (1.8%) and morbidity in 15.1% of patients. At 24 months, %total weight loss was 37.6 ± 12.3 and %excess weight loss 76.9 ± 25.2 (64 patients). Three patients were treated for malnutrition during follow-up, two with medical treatment and one with SADI reversal.

**Conclusion:**

SADI after SG provides effective weight loss results in the short-term, even if in the present series the postoperative complication rate was non-negligible. Further trials are needed to establish the more advantageous revisional bariatric procedure after failed SG.

**Supplementary Information:**

The online version contains supplementary material available at 10.1007/s11695-021-05323-y.

## Introduction

Sleeve gastrectomy (SG) represents the most performed bariatric procedure worldwide, with 340,550 surgeries occurring in 2016 according to the International Surgery of Obesity and Metabolic Disorders (IFSO) [1, 2]. SG has become the most common procedure for several reasons, which include its technical simplicity, excellent short-term weight loss, and safety profile [3, 4]. However, recent studies with longer follow-up have underlined two major disadvantages associated with SG. At 10-year follow-up, a relevant proportion of patients ranging from 10 to 50% are affected by weight regain [5–7]. Furthermore, symptoms of gastroesophageal reflux disease (GERD) are observed in up to 76% of patients at 5-year follow-up, with a reported incidence in Barrett’s esophagus of 18.8% [8]. The need for revisional procedures because of insufficient weight loss or weight regain, GERD, or other complications is reported to be 18–36% in studies with 10-year follow-up [6, 7].

Several revisional procedures are possible after SG, including conversion to Roux-en-Y gastric bypass (RYGB) [9], one-anastomosis gastric bypass (OAGB) [10], and duodenal switch (DS) [11]. Single-anastomosis duodeno-ileal bypass (SADI) has been proposed as an alternative to DS, with the advantages of a more simple procedure from a technical point of view, and a potentially reduced morbidity [12, 13]. The evaluation of revisional procedures after SG is essential since a consistent number of patients will probably need a conversion in the future.

The aim of this study is to evaluate the short- and mid-term outcomes of SADI after SG in a referral center for bariatric surgery.

## Methods

### Patient Selection

The Institutional Review Board of our institution approved the study, which is registered as IORG-IRB: IORG0009085 COS-RGDS-2019-11-001-LIAGRE-A. All patients who underwent SADI after SG between March 2015 and March 2020 were identified retrospectively from a prospective database that included all patients who underwent bariatric surgery in our department. Data were obtained from our database, computerized hospital records, and case notes as and when necessary. Data were further supplemented by contacting the patients and their general practitioners if needed.

### Preoperative Workup

Indications for primary surgery were in line with the National Health Authority (Haute Autorité de Santé, HAS) recommendations, and surgery was proposed as a second-line treatment after 6–12 months of the medical management of patients with body mass index (BMI) >35 kg/m^2^ and one or more obesity-related comorbidity or BMI >40 kg/m^2^ [14]. Preoperative workup included upper gastrointestinal (GI) endoscopy, abdominal ultrasound, clinical, biochemical, nutritional, and psychological assessment. The multidisciplinary obesity board of the institution validated the indication for surgery. Indications for secondary surgery were insufficient weight loss/weight regain after primary surgery (which consisted of SG), or persistent morbid obesity with BMI >35.

### Surgical Technique: SG

The technique used for SG was standard. The stomach was completely mobilized with transection of the short and posterior gastric vessels. A 36-Fr bougie was inserted into the stomach and gastric longitudinal resection starting 6 cm proximal to the pylorus was performed.

### Surgical Technique: SADI

After duodenal stapling, the ileum was measured to count a common limb of 250 cm or 300 cm according to the residual BMI. Duodenal dissection was carefully undertaken due to its fragility and the proximity to noble anatomical elements (gastroduodenal artery, pancreas, and common bile duct). A hand-sewn termino-lateral duodeno-ileal anastomosis was fashioned with a single-layer barbed suture. An antireflux procedure was associated to the SADI in case of hiatal hernia.

One experienced surgeon (who had performed more than 7000 bariatric procedures at the beginning of the present series, and received specific training) performed all the SADIs.

### Postoperative Outcomes and Follow-up

Postoperatively, water intake was started the evening of surgery and a semi-liquid diet was allowed on postoperative day 1. Computed tomography with oral contrast ingestion was systematically performed before discharge. Postoperative complications were classified according to the Clavien-Dindo classification [15]. Proton pump inhibitors (PPIs) were prescribed for 3 months after surgery. After this period, the PPI was continued only in response to GERD symptoms. Micronutrient supplementation was administered routinely to all patients after SADI. Supplementary Table 1 reports our protocols of vitamin and micronutrient supplementation after surgery.

Weight loss outcomes were expressed as percentage total weight loss (%TWL) and percentage excess weight loss (%EWL), calculated as [initial weight − follow-up weight] × 100, and [initial weight − follow-up weight] × 100 / [initial weight − ideal weight], respectively. Ideal weight was set as that equivalent to a BMI of 25 kg/m^2^. Total %TWL after SADI was calculated using the weight before SG as the initial weight, whereas additional %TWL was calculated using the weight before SADI as the initial weight. Follow-up continued with clinical and biochemical assessment at 1, 3, 6, 12, 18, and 24 months postoperatively, and once a year thereafter.

The evolution of obesity-related comorbid conditions was assessed according to the use and discontinuation of medication postoperatively in the instance of diabetes, hypertension, dyslipidemia, and osteoarthritis. Remission of hypertension was defined as a systolic blood pressure of less than 130 mmHg or diastolic blood pressure of less than 85 mmHg without the use of antihypertensive drugs. Improvement was defined as a decrease in the quantity or dosage of antihypertensive drugs. Diabetes remission was defined as fasting glucose of less than 5.6 mmol/L and a glycosylated hemoglobin value of less than 6.5% without the use of oral hypoglycemic medications or insulin. Improvement was defined as a decrease in the quantity or dosage of oral hypoglycemic medications or insulin. Improvement of osteoarthritis was evaluated based on symptoms, mobility, and use of painkillers. The presence of preoperative sleep apnea syndrome was quantified by sleep studies and postoperative resolution by discontinued use of continuous positive airway pressure masks. GI and endocrinological complications included diarrhea, hypoglycemia, abdominal pain, and GERD. Biliary reflux was defined as the presence of clinical symptoms necessitating treatment, such as heartburn and/or bile vomiting and/or biliary regurgitation, particularly during the night or in dorsal decubitus.

### Data Presentation and Statistical Analysis

Continuous data are reported as means, standard deviations, and ranges. Nominal data are expressed as numbers and percentages. Comparisons were made using the *χ*^2^ test for nominal data or Student’ *t* test for continuous data. Paired Student’ *t* test was used to compare preoperative and postoperative biochemical values. A *p* value of ≤0.05 was considered to be statistically significant. All statistical analyses were performed using SPSS software version 25.

## Results

### Patient Characteristics and Surgical Procedures

**Fig. 1:**
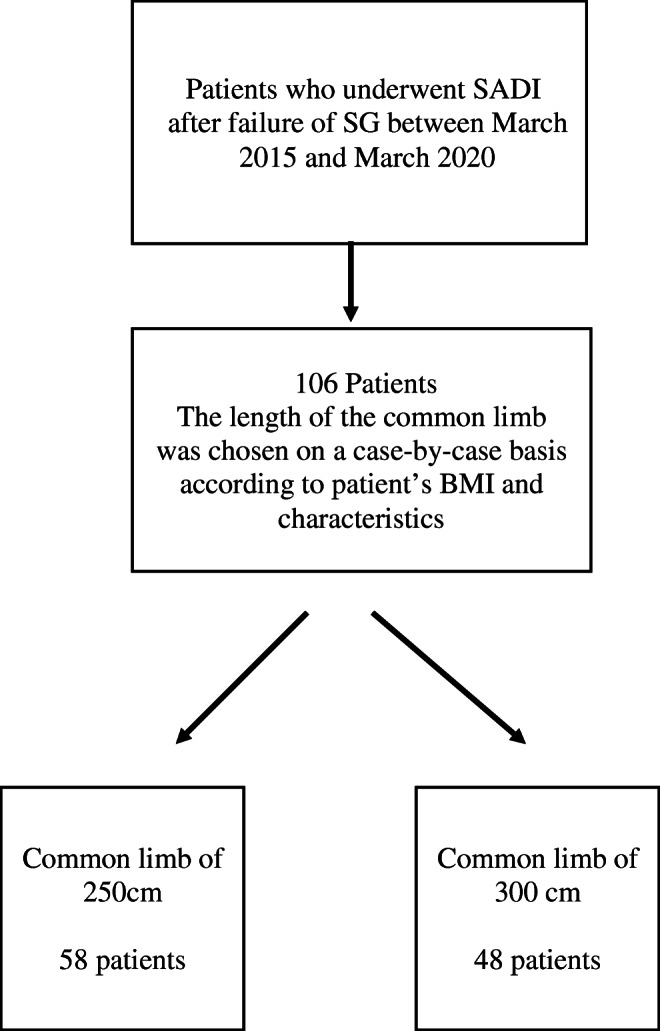
Flowchart of the included patients

During the study period, 106 patients underwent SADI after a previous SG (Fig. 1). Other previous bariatric surgery had been performed in 16 cases, including 13 adjustable gastric bandings and three re-SGs. Fifty-eight patients underwent SADI with a common limb of 250 cm and 48 with a limb of 300 cm (Fig. 1), according to their initial BMI. The characteristics and comorbidities of patients are listed in Table 1 and Supplementary Table 3, respectively. The timeframe between SG and SADI was 50 ± 31.3 months (range = 9–147). Seven (6.6%) patients had a higher weight at the time of SADI than before SG. Thirty-five (33%) patients had symptoms of GERD before SADI. Surgical procedures associated to SADI were antrectomy in one case (0.9%), hiatal hernia repair and Hill gastropexy in 26 (24.5%), resection of the gastric fundus in four (3.7%), cholecystectomy in 53 (50%).

**Table 1:** Characteristics of the included patients

**Variable**	**Before SG (*n*** **= 106)**	**Before SADI (*n*** **= 106)**
Female sex	80 (75.5%)	80 (75.5%)
Age (years)	-	46.2 ± 11.7 (21–70)
Weight (kg)	136.6 ± 29.3 (85–222)	114 ± 22.2 (76–190)
BMI (kg/m^2^)	49.8 ± 9.1 (32–75)	41.5 ± 6.1 (30–58)
%EWL > 50 after SG	-	67 (63%)
**Weight loss outcomes after SG**		
Variable		
Minimal weight	100.1 ± 23.9 (53–153)	
Higher %EWL	57.1 ± 23.3 (7–107)	
Higher %TWL	26.2 ± 10.3 (0–50)	
Lower BMI	36.5 ± 7.6 (21–55)	
Residual %EWL	30.7 ± 23.2 (−50 to 71)	
Residual %TWL	15.3 ± 11.5 (−26.4 to 37.8)	

Data are presented as absolute number (percentage) or as mean ± standard deviation (range)

*N*, number; *SG*, sleeve gastrectomy; *SADI*, single-anastomosis duodeno-ileal bypass; *BMI*, body mass index; *EWL*, excess weight loss, *TWL*, total weight loss

### Postoperative Complications

Postoperative mortality was observed in two cases (1.8%). One patient had an anastomotic leak, which caused an abscess confined to the perianastomotic region. Sudden death occurred during hospitalization and pulmonary embolism or a cardiac cause was suspected. The second has been discharged and experienced sudden abdominal pain and fever, with rapid deterioration and death at home.

Postoperative morbidity occurred in 15.1% of patients and is detailed in Table 2. Postoperative sepsis was diagnosed in 13.2% of patients and abdominal abscesses with or without an anastomotic leak in 8.4%. Patients who underwent SADI between 2015 and 2017 had a rate of abdominal abscesses with or without an anastomotic leak of 14.2% (7/49), significantly higher than the rate of those operated on between 2018 and 2020, which was 3.5% (2/57), showing a “learning-curve” effect (*p* = 0.0482). The rate of post pyloric abscess ± fistula was 1/27 (3.7%) when no associated procedures were performed. If cholecystectomy was associated to SADI, the rate of abscess ± fistula was 7/53 (13.2%) versus 2/53 (3.8%) in patients with no associated cholecystectomy (*p* = 0.0829).

**Table 2:** Postoperative complications after single-anastomosis duodeno-ileal bypass

Postoperative morbi-mortality	*N* (%)	Type of complication and treatment
Sepsis	14 (13.2%)	
Abdominal abscess ± leak	9 (8.4%)	Anastomotic leak with a 6-cm abscess, sudden death at POD 5
		Abdominal pain and fever at POD 20 with sudden death at home
		Re-laparoscopy at POD 6, with placement of a Kehr tube into the orifice of the leak. Percutaneous drainage of a hepatic abscess, drainage of a pelvic abscess
		Re-laparoscopy at POD 2, with placement of a Kehr tube into the orifice of the leak
		Re-laparoscopy at POD 2 with washing and drainage. Placement of an endoscopic Kehr tube into the orifice of the leak for chronic duodeno-cutaneous fistula
		Leak and torsion of the ileal anastomoti at POD 8. Laparotomy and conversion to OAGB
		Re-laparoscopy at POD 6, with placement of a Kehr tube into the orifice of the leak
		Diagnosis at POD 20 of a small leak by CT scan and choledocoduodenal fistula at endoscopy, treated by antibiotics
		Posterior leak at POD 10 with gastroduodenal artery erosion treated by laparoscopy, endoscopy, and embolization. Complicated by anastomotic stenosis
Perianastomotic cellulitis without abscess	4 (3.7%)	Antibiotic treatment
Bowel iatrogenic perforation	1 (0.9%)	Re-operation
Anastomotic bleeding	1 (0.9%)	Erosion of the gastroduodenal artery due to an anastomotic leak
Anastomotic stenosis	1 (.09%)	Spontaneous resolution
Total morbidity	16 (15.1%)	

*POD*, postoperative day; *OAGB*, one-anastomosis gastric bypass; *CT*, computed tomography

If gastric or hiatus procedures were associated to SADI, it was 1/27 (3.7%), versus 8/79 (10.1%) when no gastric or hiatus procedures were performed (*p* = 0.3036). Secondary procedures had a leak rate of 8/90 (8.8%) versus 1/16 (6%) for tertiary procedures (*p* = 0.7284).

### Long-term Outcomes

Compliance to vitamin treatment was observed in 71 (87.6%) out of 81 patients with follow-up longer than 12 months. Two patients were hospitalized for malnutrition during follow-up and underwent medical treatment, whereas one patient underwent SADI reversion for malnutrition, chronic diarrhea, and abdominal pain. Two patients needed intravenous iron injections. Nutritional blood test results are reported in Supplementary Table 5. Weight loss outcomes after SADI are reported in Table 3. Postoperative GERD was present in 19 (17.9%) patients and was treated medically in 15 cases, with surgery in four. Episodes of hypoglycemia were diagnosed in six (5.6%) patients, who underwent medical treatment; diarrhea occurred in 11 (9.4%) patients, of whom one underwent surgical revision. Long-term complications and additional procedures are reported in Table 4.

**Table 3:** Weight loss outcomes after single-anastomosis duodeno-ileal bypass

	12 months	24 months	36 months	48 months
Weight	88.5 ± 19.2 (54–145)	87.8 ± 20.7 (53–139)	95.8 ± 18.4 (62–134)	94.7 ± 13.3 (75–113)
BMI	32.2 ± 6.9 (22–46)	31.8 ± 6.7 (20–57)	34.5 ± 6.4 (26–55)	33.9 ± 3 (29–38)
%EWL	74.1 ± 22.1 (10–141)	76.9 ± 25.2 (18–160)	68.5 ± 22.7 (18–97)	78.2 ± 9.4 (68–95)
%TWL (overall)	36 ± 10.5 (4.5–58.9)	37.6 ± 12.3 (10.9–63.8)	35.6 ± 12.6 (7.5–57.8)	44.1 ± 8.2 (32.1–59.4)
Additional %TWL	22 ± 9.1 (2.4–48.1)	23.2 ± 12.9 (−9 to 53.4)	19.4 ± 12.7 (−4.6 to 38.7)	22.6 ± 9.6 (6.9–36.6)
*N*. of patients	81	64	31	10
Lost to follow-up	1	2	1	1

*BMI*, body mass index; *EWL*, excess weight loss; *TWL*, total weight loss; *N.*, number

**Table 4:** Long-term complications and additional procedures in patients with follow-up longer than 12 months after SADI (*N* = 81)

Complication/additional procedure	Number (percentage)
Cholecystectomy	6 (7.4%)
Urinary lithiasis	5 (6.2%)
GERD managed with medical treatment	15 (18.5%)
Invalidating reflux treated by conversion to RYGB	1 (1.2%)
Invalidating reflux treated by hiatal hernia repair and gastropexy	3 (3.7%)
Anastomotic stenosis	0
Re-SG for insufficient weight loss	2 (2.5%)
Malnutrition managed with medical treatment	2 (2.5%)
Laparoscopy for abdominal pain	1 (1.2%)
Conversion to RYGB for abdominal pain, diarrhea, and malnutrition	1 (1.2%)
Internal hernia	0
Cerebral hematoma	1 (1.2%)
Incisional hernia/umbilical hernia	1 (1.2%)
Gastric antrum resection for insufficient weight loss	1 (1.2%)
Duodenal ulcer	1 (1.2%)

*SADI*, single-anastomosis duodeno-ileal bypass; *SG*, sleeve gastrectomy; *RYGB*, Roux-en-Y gastric bypass

Supplementary Table 8 reports the outcomes according to weight loss obtained after SG (%EWL at 24 months follow-up). Interestingly, no significant differences were found in TWL at 12 and 24 months after SADI between the two groups.

Table 5 reports the outcomes of SADI with a 250-cm versus 300-cm common limb. The evolution of comorbidities is shown in Supplementary Table 3. Patients undergoing SADI with a 250-cm common limb had higher BMI before SG and before SADI. They also had significantly higher %TWL at 24 months follow-up after SADI. The rate of postoperative complications was comparable.

**Table 5:** Comparison between patients with a 250-cm common limb and those with a 300-cm common limb

	Common limb = 250 cm	Common limb = 300 cm	*p*
	58	48	
Weight at the time of SG (kg)	151.6 ± 27.9 (87–222)	118 ± 20 (85–185)	<0.0001
BMI at the time of SG (kg/m^2^)	54.3 ± 8.4 (33–75)	44.2 ± 6.6 (32–63)	<0.0001
Weight at the time of SADI (kg)	124 ± 23 (79–190)	102 ± 13.9 (76–138)	<0.0001
BMI at the time of SADI (kg/m^2^)	44.3 ± 5.8 (34–58)	38 ± .5 (30–51)	<0.0001
Weight 12 months after SADI (kg)	93.3 ± 20.2 (54–145)	81 ± 14.7 (57–115)	0.0048
BMI 12 months after SADI (kg/m^2^)	33.5 ± 6 (22–46)	30 ± 5 [16–37]	0.0101
%EWL 12 months after SADI	72.9 ± 19.4 (32–141)	75.9 ± 26 (10–124)	0.5598
%TWL 12 months after SADI	38 ± 9.5 (18–58.9)	32.8 ± 11.4 (4.5–56.7)	0.0304
Additional %TWL at 12 months after SADI	23.4 ± 9.4 (3.2–58.9)	19.8 ± 8.4 (2.4–42.3)	0.0884
Weight 24 months after SADI (kg)	91.6 ± 21.3 (54–139)	81 ± 18 (53–120)	0.0492
BMI 24 months after SADI (kg)	33 ± 7.2 (20–57)	29.4 ± 5.1 (22–39)	0.0607
%EWL 24 months after SADI	77 ± 24.1 (18–160)	76.7 ± 27.5 (27–128)	0.9687
%TWL 24 months after SADI	40.4 ± 11.5 (10.9–63.7)	32.5 ± 12.1 (11.1–59.4)	0.0067
Additional %TWL 24 months after SADI	25.5 ± 12.8 (−8.5 to 53.3)	18.9 ± 12.2 (−9 to 40.9)	0.0351
Sepsis	15.5%	10.4%	0.5678
Postoperative death	3.4%	2%	0.1961
Hospitalization for malnutrition	3.4% (*n* = 2/medical treatment)	2% (*n = *1/surgical treatment)	0.8331
% of patients with normal pre-albumin 12 months after SADI	67.5% (*n* = 23/34)	69.5% (*n* = 16/23)	0.1437

*SG*, sleeve gastrectomy; *SADI*, single-anastomosis duodeno-ileal bypass; *BMI*, body mass index; *EWL*, excess weight loss; *TWL*, total weight loss

## Discussion

The present study demonstrates that SADI is an effective revisional bariatric procedure after failed SG. However, a non-negligible rate of postoperative morbidity and mortality was observed in the present series, in comparison with previous published series on other revisional procedures after SG and other series of revisional SADI [10, 11, 38, 39]. SADI is effective in patients with insufficient weight loss, or it may be scheduled after SG in patients with a very high BMI, in the setting of a two-step surgical strategy.

One of the tendencies of bariatric surgery is toward simplification of the procedures. In this setting, one-anastomosis procedures have been developed, OAGB as an alternative to RYGB, SADI as an alternative to DS [40–43]. SADI has been proposed as a more “simple” version of the DS by Sanchez-Pernaute and Torres [12, 13]. The main characteristic of one-anastomosis bariatric surgeries is the flow of a high volume of bile and intestinal content into a single anastomosis [13]. In this setting, a leak of the anastomosis may cause severe peritonitis, due to a large amount of fluid spilling from the anastomosis. When leaks develop after OAGB, the spillage occurs in a confined space in the majority of cases, between the liver and the residual stomach, and non-surgical treatment may be effective [16], whereas after SADI, generalized peritonitis is more frequent in our experience.

Concerning the surgical technique, SADI after SG may or may not include a re-sleeve of the gastric tube. Re-sleeve is associated with a higher rate of postoperative leaks, which is estimated to be around 2% [17], compared to primary SG [18]. To avoid a potential source of additional postoperative complications, in our center, we avoid re-sleeving the gastric pouch during SADI. When we perform OAGB after SG, we recalibrate the gastric tube systematically, increasing the restriction effect of the primary procedure [10]. In the absence of re-SG, the effect of secondary surgery relies only on the malabsorption effect; however, this may also be responsible for malnutrition.

SADI as a revisional procedure after SG is undoubtedly effective on weight loss outcomes in the present series, leading to an additional %TWL of 23.2 at 24 months (data referring to 64 patients). These results seem to last for 48 months of follow-up, even if available for a minority of patients. Other authors report comparable short-term outcomes, whereas longer follow-up is needed to verify the durability of the weight loss (Table 6). Among the previous series, Sanchez-Pernaute et al. [19] demonstrated the efficacy of SADI in a population of 97 diabetic patients, achieving control of the disease in 70–84% of patients long-term, and %EWL of 98 at 5-year follow-up. SADI was effective also in improving the patients’ quality of life [20]. A large series by Finno et al. [21] compared 259 patients undergoing DS with 181 who had SADI-S (single-anastomosis duodeno-ileal bypass with sleeve gastrectomy). The authors reported comparable results in terms of postoperative morbidity (13.3% after SADI-S versus 18.9% after DS), with results comparable to those of the present series. Two-year weight loss outcomes were comparable, too. However, a late complication rate and vitamin and micronutrient deficiencies were higher after DS. Surve et al. [22] analyzed the results of 750 primary SADI-S, showing a morbidity rate of 7.8% and effective weight loss results at 60 and 72 months of follow-up.

**Table 6:** Results of previous series reporting revisional procedures after SG

Author, year	*N*.	Procedure	Morbidity	%TWL	%EWL	Follow-up	*N*. f-u
Debs 2020	77	OAGB	3.9%	25	74	12 months	70
				29	79	24 months	56
				26	77	60 months	32
Jamal 2020	56	OAGB	0%	28.8	84.9	12 months	27
Bashah 2020	42	SADI	19%*	23.7	57.6	12 months	-
				26.4	65.8	18 months	-
	49	OAGB	27%*	18.7	47.1	12 months	-
				21.2	52.1	18 months	-
Alsabah 2018	31	OAGB	10.3%	-	58.9	12 months	-
Dijkhorst 2018	66	SADI	16.7%**	21.5		12 months	-
				26.4		24 months	-
	74	RYGB	17.6**	8.9		12 months	-
				6.9		24 months	-
Andalib 2020	41	RYGB	14.6%	10.1	27.6	12 months	33
	33	DS	3%	14	31.6	12 months	25
	7	SADI	0%	9.4	55.1	12 months	3
	13	re-SG	0%	7.6	29.2	12 months	10
Sanchez-Pernaute	51	SADI	-	39	79	12 months	41
				41	81	24 months	29
				38	76	36 months	21
				41	80	48 months	17
				41	79	60 months	17

*Including long-term complications; **including complications within the first year

*SG*, sleeve gastrectomy; *N.*, number; *TWL*, total weight loss; *EWL*, excess weight loss; *N. f-u*, number of patients available at follow-up, *OAGB*, one-anastomosis gastric bypass; *SADI*, single-anastomosis duodeno-ileal bypass; *RYGB*, Roux-en-Y gastric bypass; *DS*, duodenal switch

The IFSO has supported SADI-S as a recognized bariatric/metabolic procedure encouraging further studies to better elucidate its long-term efficacy and safety [23]. The American Society for Metabolic and Bariatric Surgery (ASMBS) endorsed SADI as an appropriate bariatric procedure, encouraging more studies on long-term results [24].

Postoperative morbidity occurred in 15.1% of patients in the present study, with a sepsis rate of 13.2% and abdominal abscess with or without leak of 8.4%. Mortality occurred in two patients. In keeping with other studies, there were no internal hernias in this group. The rate of postoperative complications was higher in the present series of SADI as a revisional procedure than after primary SADI in other published series. The multicenter study by Surve et al. [25] including 1328 patients reported a low morbidity rate, which included anastomotic leak in 0.6% of cases, ulcer in 0.1%, stricture in 0.3%, and bile reflux in 0.1%. The study about primary SADI published by Zaveri et al. reported a rate of 7.7% of postoperative complications and 0.2% of postoperative mortality [26]. The morbidity and mortality rate of the present series was also higher compared to the one reported by Zaveri et al. after SG, as the latter authors had a postoperative early and late complication rate of 5.3% and 6.4%, respectively [38]. We underline that in the present series 15.1% of patients underwent two bariatric procedures before SADI, which was therefore a third surgery in these cases. Furthermore, concomitant procedures, mostly cholecystectomy, were performed in the majority of our patients. These characteristics of our population of study may explicate the higher morbidity and mortality rate compared to the series by Zaveri et al. [38]. It should also be remarked that the morbidity rate was significantly related to the period of surgery, showing a role of the “learning-curve”: abscess and/or leak rate dropped from 14.2% in the first 3 years to 3.5% in the second 3-year period in the present study.

Other series of SADI performed as a second-step procedure reported complication rates of around 15% [27–29], or lower [11, 38]. In our center, SADI after SG had higher short-term postoperative morbidity and mortality than OAGB after SG [10]. On the other hand, de la Cruz et al. reported similar outcomes in terms of morbidity and weight loss after OAGB and SADI as revisional procedures after SG [39].

Table 6 reports the published series of revisional bariatric surgery after the failure of SG. All these series include a relatively low number of patients, and there is no evidence to recommend one procedure over the other after the failure of SG [11, 28–32]. A recent meta-analysis showed a revision rate of 10.4% after SG, rising to 22.6% for patients with follow-up longer than 10 years [33]. The most common revisional procedure was RYGB, even if the results of different procedures were not compared.

SADI is characterized by some important technical points, advantages, and disadvantages that should be underlined. The duodeno-ileal anastomosis is in our opinion more technically demanding than the gastrojejunal anastomoses of RYGB and OAGB. Duodenal tissue is thinner and more fragile than that of the gastric wall. Furthermore, in anastomotic leaks, the spillage of intestinal fluid and bile is frequently responsible for generalized peritonitis, due to the location of the anastomosis. The length of the common limb is another debated technical topic and is of maximal importance for its relationship with both the efficacy of weight loss and the rate of nutritional complications [34–37, 44–46]. In the present series, three patients have been treated for severe malnutrition (two with medical treatment, one with surgical revision). The analysis of biochemical values before and after SADI showed a very high rate of preoperative vitamin D deficits, which was only partially ameliorated after surgery. The rate of hyperparathyroidism was not negligible, with abnormal values in 49.1% of patients at 12 months of follow-up and 63.3% at 24 months, which may represent a risk of osteopenia [47]. We recommend prompt vitamin D supplementation starting before surgery to minimize serious deficits and potential complications.

SADI after SG does not overcome the disadvantages of SG, including the potential development of GERD, and possible gastric tube dilatation, with subsequent loss of restriction. On the other hand, the preservation of the pylorus may carry some advantages, such as a reduced rate of anastomotic ulcer and less hypoglycemic episodes and dumping. It is not clear if SADI may be avoided in patients with significant preoperative GERD, in the light of recent data about GERD and Barrett’s esophagus after SG [8]. However, in our opinion, preoperative assessment of the hiatus to search for hiatal hernia is very important; the hiatal hernia should be searched for during surgery and, if found, repaired.

Concerning cholecystectomy, half of the patients received it during SADI, and in the remnant patients, it was needed in 7.4% during follow-up. Cholecystectomy after a previous SADI may be more challenging due to potential adhesions following duodenal dissection. In the present series, the rate of abdominal abscess and/or leak of patients undergoing SADI with synchronous cholecystectomy was 13.2%, higher (however without statistical significance, which may be due to the number of included patients limiting the statistical power) than the one of patients undergoing SADI without additional procedures (3.7%) or with synchronous gastric or hiatus procedure (3.7%). Therefore, we recommend avoiding synchronous cholecystectomy during SADI.

We also underline that long-term complications requiring additional surgery were relatively infrequent if we compare the present series with historical series of RYGB [48].

Concerning long-term outcomes of SADI, a relevant study recently reported data from 750 patients with 5 years of follow-up available on 109 patients (61%) and at 6 years on 87 patients (53%) [22]. The long-term complication rate was 11.7%, including diarrhea, nausea and vomiting, strictures, constipation, cholelithiasis, GERD, retrograde filling of the afferent limb, malnutrition, dilated fundus, gastric outlet obstruction, and hiatal and ventral hernia [22].

After SADI, patients require a strict follow-up, to minimize and promptly treat long-term complications. During the first year after surgery, we recommend clinical follow-up every 3 months, and every 6 months thereafter. Patients should be followed by a bariatric unit (including surgeons and physicians specialized in nutrition) and a general practitioner. The present study underlines that the association of SADI with synchronous cholecystectomy is associated with a trend for a higher risk of postoperative morbidity; therefore, we recommend, if it is possible and after careful clinical evaluation, avoiding cholecystectomy during SADI.

## Conclusion

SADI after SG provides effective weight loss results in the short-term, at the cost of a non-negligible rate of postoperative and mid-term morbidity in the present series, in comparison with previous published series on other revisional procedures after SG and other series of revisional SADI. Further trials are needed to establish more advantageous revisional bariatric procedures after failed SG.

## Supplementary Information

ESM 1 (DOCX 19 kb)

ESM 2 (DOCX 62 kb)

ESM 3 (DOCX 136 kb)

ESM 4 (DOCX 123 kb)
