# Supplementary material for: Efficacy and Drawbacks of Single-Anastomosis Duodeno-Ileal Bypass After Sleeve Gastrectomy in a Tertiary Referral Bariatric Center
Source: Obes Surg. 2021 Apr 9;31(6):2691–700. doi: 10.1007/s11695-021-05323-y (PMC8113294; doi:10.1007/s11695-021-05323-y)
Supplement: Supplementary file 1 — (DOCX 19 kb) [file 11695_2021_5323_MOESM1_ESM.docx]

**Table 1 (supplementary). Protocols of vitamins and micronutrients supplementation for patients undergoing single anastomosis duodeno-ileal bypass in our institution**

| Supplementation from 2013 to 2018 | Supplementation since 2018 |
| --- | --- |
| **Azinc optimal** **2/day**  Vitamin A 800μg  Vitamin B1 1,4mg  Vitamin B2 1,6mg  Vitamin B3 18mg  Vitamin B5 6mg  Vitamin B6 2mg  Vitamin B8 150μg  Vitamin B12 1μg  Vitamin C 120mg  Vitamin D3 5μg  Vitamin E 10mg  Chromium 25μg  Copper 1,5mg  Iron 8mg  Manganese 3,5mg  Molybdenum 80μg  Selenium 50μg  Zinc 15mg  **Caltrade Vit D3** **2/day**  Calcium 1200mg,  Vitamin D3 800UI  **Vit B12 1/7 days**  Vitamin B12 1000 µg  **Uvedose** **1/30jour**  Vitamin D3 100 000UI  **Tardyferon** **1/jour**  Iron 80mg | **WLS Maximum 1/day FitForMe**  Vitamin A 1200μg  Vitamin B1 3mg  Vitamin B2 3,5mg  Vitamin B3 32mg  Vitamin B5 18mg  Vitamin B6 1,4mg  Vitamin B8 100μg  Vitamin B11 800μg  Vitamin B12 500μg  Vitamin C 120mg  Vitamin D3 75μg  Vitamin E 20mg  Vitamin K1 300μg  Chromium 160μg  Copper 4mg  Iron 91mg  Iodine 150μg  Manganese 3mg  Molybdenum 112μg  Selenium 105μg  Zinc 30mg  **Calcium Forte** **1/jour**  Calcium 1000mg  Vitamin D3 25μg |
