# Supplementary material for: Efficacy and Drawbacks of Single-Anastomosis Duodeno-Ileal Bypass After Sleeve Gastrectomy in a Tertiary Referral Bariatric Center
Source: Obes Surg. 2021 Apr 9;31(6):2691–700. doi: 10.1007/s11695-021-05323-y (PMC8113294; doi:10.1007/s11695-021-05323-y)
Supplement: Supplementary file 2 — (DOCX 62 kb) [file 11695_2021_5323_MOESM2_ESM.docx]

Table 3 (Supplementary). Comorbidities and their evolution after single anastomosis duodeno-ileal bypass

| Comorbidity | % before SADI (n=) | > 12months after SADI (n=94) | Rate of resolution |
| --- | --- | --- | --- |
| Arterial hypertension | **32%**  (34/106) | Resolution = 14  Not resolved = 14  Not available = 6 | **50%** |
| Diabetes | **20%**  (21/106) | Resolution = 18  Not resolved = 2 | **90%** |
| OSAS | **23%**  (24/106) | Resolution = 13  Not resolved = 8  Not available = 3 | **65%** |
| Rheumatologic comorbidities | **14%**  (15/106) | Improvement = 11  Not resolved = 2  Not available = 2 | **85%** |

OSAS, obstructive sleep apnea syndrome; SADI, single anastomosis duodeno-ileal bypass
