# Supplementary material for: Efficacy and Drawbacks of Single-Anastomosis Duodeno-Ileal Bypass After Sleeve Gastrectomy in a Tertiary Referral Bariatric Center
Source: Obes Surg. 2021 Apr 9;31(6):2691–700. doi: 10.1007/s11695-021-05323-y (PMC8113294; doi:10.1007/s11695-021-05323-y)
Supplement: Supplementary file 3 — (DOCX 136 kb) [file 11695_2021_5323_MOESM3_ESM.docx]

Supplementary Table 5. Blood tests results before and at 12 and 24 months after single anastomosis duodeno-ileal bypass

| Biochemical variables  (n=106) | Preoperative values | % of abnormal results | 12 months after SADI | % of abnormal results at 12 months | 24 months after SADI | % of abnormal results at 24 months | P* |
| --- | --- | --- | --- | --- | --- | --- | --- |
| Hemoglobin  (12-16 g/L) | 13.5±1.4  (9.2-17)  (n=104 ) | **12.5%** | 13.2±1.5 (8.7-16.8)  (n=60 ) | **13.4%** | 13.2±1.38 (10-16.7  (n=46 ) | **17.4%** | 0.1042 |
| Albumin  (35-52 g/L) | 40±2.9  (32-46)  (n=98 ) | **2%** | 38.5±4.5 (20-47)  (n=58 ) | **15.6%** | 38.9±3.5  (32-45)  (n=44 ) | **6.9%** | 0.0631 |
| Ferritin  (15-150 μg/L) | 87±94.5  (6-623)  (n=101 ) | **12.5%** | 108.7±98.2 (4-389)  (n=59 ) | **10.2%** | 137 ±189 (5-1141)  (n=45 ) | **15.6%** | 0.1010 |
| Prealbumin  (0.2-0.4 g/L) | 0.26±0.04  (0.15-0.38)  (n=99 ) | **9.1%** | 0.22±0.05 (0.09-0.36)(  n=57 ) | **31.6%** | 0.22±0.05 (0.1-0.32)  (n=43 ) | **28%** | 0.0053 |
| Vitamin A  (1.72-2.52 μmol/L) | 1.79±0.39  (1.19-2.48)  (n=20 ) | **60%** | 1.6±0.6 (0.27-3.7)  (n=49 ) | **61.3%** | 1.58±0.46 (0.88-2.68)  (n= 38) | **60.6%** | 0.1001 |
| Vitamin B9  (10-79 ng/L) | 19.1±27.6  (4-231)  (n=88 ) | **38.7%** | 34.5±27.1 (2.4-113)  (n=57 ) | **19.3%** | 43.7± 37.3  (4.5-156)  (n= 46) | **17.4%** | 0.0122 |
| Vitamin B12  (145-569 pmol/L ) | 319.7±131.6  (50-819)  (n=99 ) | **4%** | 424±134 (150-746)  (n= 58) | **0%** | 481.1± 165 (178-908)  (n=43 ) | **0%** | <0.0001 |
| Vitamin D  (75-150 nmol/L ) | 65±53  (11-512)  (n=99) | **68.8%** | 69±22.5 (20-140)  (n=57 ) | **61.5%** | 68.7±22.4 (23-113)  (n=45 ) | **60%** | 0.3790 |
| Parathyroid hormone  (6-50 pg/mL) | 58±28.6  (18-133)  (n=17 ) | **35.3%** | 55.8± 30.4 (5-161)  (n=53 ) | **49.1%** | 67.2±32.7 (9-133)  (n=38 ) | **63.3%** | 0.0454 |
| Calcium  (2.15-2.5 mmol/L) | 2.35±0.09  (2.08-2.63)  (n=102 ) | **2%** | 2.28±0.11 (1.82-2.49)  (n= 58) | **10.4%** | 2.28±0.1 (2.1-2.4)  (n= 45) | **5%** | 0.0003 |

* p values are calculated comparing preoperative values with values at 24 months follow-up
