# Supplementary material for: Efficacy and Drawbacks of Single-Anastomosis Duodeno-Ileal Bypass After Sleeve Gastrectomy in a Tertiary Referral Bariatric Center
Source: Obes Surg. 2021 Apr 9;31(6):2691–700. doi: 10.1007/s11695-021-05323-y (PMC8113294; doi:10.1007/s11695-021-05323-y)
Supplement: Supplementary file 4 — (DOCX 123 kb) [file 11695_2021_5323_MOESM4_ESM.docx]

Table 8 (Supplementary). Comparison between patients %EWL≥50 and those with %EWL<50 after SG

|  | Effective SG  EWL> 50% (n=67) | Ineffective SG  EWL< 50% (n=39) | p |
| --- | --- | --- | --- |
| **Weight at the time of SADI (kg)** | 105.8±19.5 (76-190) | 128.1±19.6 (82-177) | <0.0001 |
| **BMI (kg/m^2^) at the time of SADI** | 38.9±4.9 (30-55) | 45.8±5.5 (34-58) | <0.0001 |
| **Weight before SG** | 129.5±28.1 | 148.8±27.5 (98-222) | 0.0008 |
| **BMI before SG** | 47.8±9 (32-72) | 53.2±8.4 (39-75) | 0.0028 |
| **Minimal weight after SG** | 87.6±17 (53-127) | 121.6±18 (81-175) | <0.0001 |
| **Maximal %EWL after SG** | 70.6±17.5 (50-119) | 34±9.9 (7-47) | <0.0001 |
| **Maximal %TWL after SG** | 31.5±8.3 (0-50) | 17.5±6.8 (1-27.5) | <0.0001 |
| **Minimal BMI after SG** | 32.3±5.4 (21-45) | 43.6±5.2 (33-55) | <0.0001 |
| **Residual %EWL after SG** | 35.2±22 (-39-71) | 23±23.4 (-60-47) | 0.0089 |
| **Residual %TWL after SG** | 16.8±11.1 (-10-37.8) | 12.73±11.7 (-23.4-40) | 0.0771 |
| Lost to follow-up at 12 month after SADI | 0/53 (**0%)** | 2/29 (**6,8%)** | 0.0544 |
| Weight at 12 months after SADI | 80.1±14.7 (54-122) | 105±16 (85-145) | <0.0001 |
| BMI at 12 months after SADI | 29.7±4.5 (22-42) | 36.8±5.5 (27-46) | <0.0001 |
| %EWL at 12 months after SADI | 82±19.9 (36-141) | 58.6±17.9 (10-93) | <0.0001 |
| %TWL at 12 months after SADI | 38.3±10 (15-59) | 31.3±10.2 (4.5-48.9) | 0.0043 |
| Additional %TWL at 12 months after SADI | 22.9±9.8 (2.4-48.1) | 20.3±7.5 (3.2-43.4) | 0.2195 |
| Lost to follow-up at 24 month after SADI | 1/43 (**2.3%)** | 2/25 (**4.6%)** | 0.2930 |
| Weight at 24 months after SADI | 79.8±17.2 (53-120) | 102.1±18.8 (72-139) | <0.0001 |
| BMI at 24 months after SADI | 29.5±5.4 (20-41) | 35.9±6.9 (25-57) | 0.0001 |
| %EWL at 24 months after SADI | 83.8±24.2 (33-160) | 64.5±22.4 (18-96) | 0.0026 |
| % TWL at 24 months after SADI | 39.6±11.8 (12.7-63.8) | 33.9±12.5 (10.9-53.7) | 0.0741 |
| Additional %TWL at 24 months after SADI | 23.4±13.1 (-90-53.4) | 22.7±12.8 (-8.5-45.2) | 0.8199 |

SG, sleeve gastrectomy; SADI, single anastomosis duodeno-ileal bypass; BMI, body mass index; EWL, excess weight loss; TWL, total weight loss
